# Supplementary material for: Are carbon emissions trading and green financial instruments synergistic? -Comprehensive quantitative research based on content analysis
Source: PLoS One. 2024 Mar 7;19(3):e0298601. doi: 10.1371/journal.pone.0298601 (PMC10919601; doi:10.1371/journal.pone.0298601)
Supplement: S1 Data — (ZIP) [file pone.0298601.s001.zip › Data/地级市Z_F工作报告.zip!/╜¡╦╒/─╧═¿╩╨/└┤╫╘╣1⁄2╓┌║┼í╛┬φ┐╦╩2╛▌═°í┐_2018.docx]

各位代表：

　　现在，我代表市人民政府向大会作工作报告，请予审议，并请政协委员和其他列席人员提出意见。

　　一、2017年工作回顾

　　2017年，我们在省委省政府和市委的正确领导下，以习近平新时代中国特色社会主义思想为指导，认真贯彻党的十九大精神，围绕推进“两聚一高”新实践、建设上海“北大门”新定位，坚定不移抓项目促发展，持之以恒惠民生防风险，较好完成全年目标任务，实现十五届市政府工作的良好开局。经济运行平稳健康，完成地区生产总值7734.6亿元，增长7.8%；一般公共预算收入590.6亿元（消化“营改增”因素，基本持平）；固定资产投资4959.2亿元，增长8.9%；社会消费品零售总额2873.4亿元，增长9.1%；进出口总额2360.2亿元，增长16%。“三大转型升级”取得重大进展，高新技术产业产值7564.3亿元，占规模以上工业比重50.3%，均居全省第二；标注南通制造新高度的“天鲲号”、“龙源振华3号”和2万标箱超级货轮先后亮相；服务业增加值增长9.4%；《南通建设上海大都市北翼门户城市总体方案》获省政府批复实施，中央创新区建设全面启动，轨道交通1号线一期工程正式开工，沪通长江大桥投资量达60%，城市和交通格局加速提升。生态环境状况积极改善，市县镇村四级河道都有了“河长”，“263”专项行动和“三河三行业”整治扎实推进，PM2.5平均浓度降至40微克/立方米、全省最低，地表水省控断面优Ⅲ比例64.5%。居民收入稳步提高，城镇居民人均可支配收入42756元，农村居民人均可支配收入20472元，分别增长8.9%和9.2%，6.23万省标以下低收入人口收入全部超过6000元。

　　回顾过去的一年，困难和挑战比预想的大，机遇和期望也比以往的多。去年4月，国务院对2016年真抓实干、成效明显的地方予以表扬激励，我市在工业转型升级、环境治理保护、土地集约节约利用等方面有8项入选。我们以此为鞭策，撸起袖子加油干，统筹兼顾抓落实。一年来，主要做了以下工作：

　　（一）坚持稳中求进，发展基础更加厚实。深化供给侧结构性改革。依法依规淘汰落后产能，开展化工“四个一批”专项行动和沿江化工园区整治，保持打击“地条钢”高压态势，完成省下达钢铁、水泥去产能任务，关闭化工、印染、钢丝绳等行业低端低效产能企业164家。采取分类调控、增加供给、规范秩序等措施，有效稳定房地产市场。支持企业去杠杆，新增直接融资624亿元；实施政府投融资体制改革，力推国有融资平台市场化转型，规范举债融资行为，制定地方债务化解方案，新增地方政府债券及置换债券330亿元。落实七大类降成本措施，仅减税降费就减轻企业负担70亿元。量质并举抓好项目建设。完善“五个一”推进机制，举行重大项目集中开工等活动，加强定期会办、及时协调、跟踪推动，紧盯实物工作量，省级重大项目、市级“双百工程”项目分别完成投资230亿元、830亿元，新开工亿元以上产业项目增长53.6%。举办江海国际博览会暨首届通商大会、海外通商峰会和台商产业转型升级峰会等活动，在谈、签约、新开工50亿元以上重特大工业项目29个，注册外资实际到账24.2亿美元，新引进超亿美元项目38个、超亿元市外民资项目160个。推动需求结构优化升级。实施十大扩消费行动，培育“互联网+商贸”，电子商务交易额增长33%。制定落实外贸稳增长政策，市场采购贸易方式、跨境电商、外贸综合服务等新业态贡献出口增量60亿元，飞机部件航空维修再制造加工贸易业务展开。

　　（二）强化创新驱动，实体经济更有质量。出台《建设国家创新型城市打造创新之都行动方案》，通过国家创新型试点城市评估，全社会研发投入占地区生产总值比重2.69%。提高科技创新能力。落实通创币、创新券等政策，新增省级以上研发机构34家，高新技术企业突破1000家。整合高新区、南通产研院等“双创”资源，规划建设众创社区，入选省首批备案试点2家。发挥大院大所对接、江海英才创业周等效应，深化产学研合作，新建技术转移中心4家。落实“人才八条”新政，制定22个配套政策和中央创新区专项政策，新增省“双创”人才61名、团队7个，均居全省前列。新增PCT专利申请量全省第二。推进产业创新转型。组建产业技术创新联盟，举办“企业家面对面”活动，“3+3”重点产业产值占规模以上工业比重60.4%。船舶海工、电子信息、高端纺织加快智能化改造，新增国家试点示范企业3家、制造业单项冠军企业3家、技术创新示范企业1家。举办新一代信息技术博览会，率先建立大数据工作体系，IP城域网出口带宽5764G。建筑业特级资质企业达20家，“鲁班奖”总数达100个。支持企业创新创业。整合财政专项资金，制定实施促进经济发展提质增效10项政策措施、“5215”工业大企业培育计划。开展江海创投行、金融帮服万企行等活动，建立融资担保风险分担、投贷联动风险补偿机制。新增省名牌企业84家，获评省商标战略实施示范市。评选表彰“张謇杯”杰出企业家、“三名”人物和“杰出通商”。

　　（三）落实战略定位，开发开放更具活力。积极策应“一带一路”建设、长江经济带发展和以扬子江城市群为龙头的“1+3”功能区战略，拓展双向开放新空间。全面对接服务上海。举办对接服务上海大会以及科技、旅游对接和农产品展销等系列活动，承接亿元以上产业项目236个，落户高层次研发服务平台6家。围绕构建“三港三城三基地”，制定9个专项实施方案，成立沪通交通对接联合工作组，深化沪通空港物流协作，城域网实现与上海直达互联，全市新增沪通产业合作平台10个。呼应崇明世界级生态岛建设，编制海永-启隆-东平城镇圈协同规划。攻坚突破改革难点。抓好陆海统筹发展综合改革，推进沿江沿海港口一体化，组建南通港集团，通海港区集装箱码头主体结构通过交工验收，通州湾港区总体规划获批；深化陆海统筹土地利用管理创新，推进村庄建设用地布局调整、工业用地先租后让等试点。探索供给侧结构性改革金融创新，打造省股权交易南通分中心，推进企业上市，陆海统筹发展基金实质性运作。绘制沿江“一张图”，沿海新开工10亿元以上产业项目22个，港口航道、江海河联运和海洋经济创新发展示范项目扎实推进。提升经济国际化水平。抓好省“走出去”先行先试改革试点，境外投资增长12.5%，实施跨国并购项目8个，3家企业进军以色列建筑市场。蝉联“中国服务外包风采城市”。出台支持开发园区创新转型发展的政策意见。口岸平台功能和通关便利化水平持续提升。

　　（四）推动布局优化，城乡发展更趋协调。成为住建部城市总体规划编制改革和“城市双修”、城市设计试点，形成全市域“多规合一”初步成果和38项专项规划。扎实推动城市和交通转型升级。推进中心城市“一主五片两重点”建设，寺街西南营历史文化街区保护修缮有序展开，中央创新区医学综合体等5个功能性项目开工，五山及沿江地区实施7项生态修复工程。跃龙路拓宽改造等十大工程竣工，啬园路快速化改造等十大工程开工。“三城同创”扎实推进，建成中心城区高速一环、宁启铁路生态廊道，市区新增绿地620公顷，全市新增造林11.1万亩。编制现代综合交通运输体系发展规划，苏通二通道等过江通道列入省级规划。59个重点交通项目完成投资101.6亿元，宁通直达动车开通。盐通铁路前期工作顺利推进，南通动车所纳入同步实施项目。稳步实施农业供给侧结构性改革。整市推进高标准农田建设，新建高标准农田67.7万亩。推进国家现代农业示范区“863”工程，建成高效设施农（渔）业10.3万亩，新增省级示范家庭农（渔）场33家、农业产业化龙头企业5家，“一村一品”示范村镇数、农产品出口额全省第一。粮食机械化烘干能力建设三年行动任务过半。优化城乡一体化体制机制。推进市区6项新型城镇化综合试点，建立财政与农业转移人口市民化挂钩机制。3个镇成为第二批全省经济发达镇改革试点镇。农村集体资产资源流转全部进场交易，农民合作社综合社、农村“两权”抵押贷款等试点有序推进。完成农村土地承包经营权确权登记颁证。培育特色小镇，重视特色田园乡村建设，3个村庄跻身省特色田园乡村首批试点。

　　（五）聚焦难题破解，生态治理更显铁腕。坚决整改中央、省环保督察反馈问题。标本兼治整改中央环保督察交办事项，持续整治41个突出环境问题，彻底解决通灵桥等区域污染。以接受省环保督察为契机，展开突出问题大排查快整治，启动“散乱污”企业综合整治，责令整改企业341家。扎实抓好水环境等突出问题整治。推进河岸共治，通过省级“河长制”验收，统筹落实“断面长制”，实施水污染治理项目210个，关闭禁养区内畜禽养殖场703家。完成548个大气治理项目，空气质量优良天数比例72.9%。垃圾分类和治理“六个一”行动有序开展。深入推进长江“共抓大保护”行动。制定实施长江流域（南通段）生态环境保护工作方案，对沿江新上化工项目实行“三个一律不批”，扎实开展水上过驳作业、沿江船舶和取水口、排污口等专项治理，彻底整治长期存在的53个非法码头。强化生态红线区域保护和管理，率先开展生物多样性调查，实施60个重大生态保护与修复工程。配合国家海洋督察，推进海洋生态文明示范区建设。持续深化污染减排措施。坚持总量和强度“双控”，完成节能减排目标任务。新增城镇污水管网185公里、危废年处置能力3.5万吨，83个循环经济重大项目完成投资81.7亿元。加快生态文明改革措施落地。出台生态环境保护工作责任规定、工业企业资源利用绩效综合评价办法，开展战略环评、生态环境损害赔偿、污染物排放许可等试点，积极发展绿色金融，建立覆盖全市的水环境区域补偿制度。

　　（六）突出精准发力，民生保障更好完善。实施富民增收“五大行动”，推进基层基本公共服务功能配置标准化，民生支出占一般公共预算支出的77%，完成11件50项重点民生实事。推进富民增收和精准脱贫。着力促进农民增收，支持返乡人员创业，发放创业担保贷款2.2亿元、稳岗补贴1.1亿元，城镇登记失业率1.82%、新增就业8.7万人。推进社会救助制度与扶贫开发政策衔接，实施健康扶贫工程，年收入7000元以下的建档立卡低收入人口脱贫率64.75%。老区建设持续加强。健全社会保障制度。五大基本社会保险新增参保29.1万人。调整完善职工生育保险等政策，城乡居民医保整合到位，企业退休人员养老金、城乡低保、特困人员供养等标准继续提高。医养、医护结合的照护保险覆盖120万人，新增护理型养老机构10家、床位1687张。“救急难”制度进一步完善。加快教育现代化市级示范区建设。优化中小学、幼儿园布局，推进城乡义务教育一体化发展，加快普通高中特色学校建设，4所高校入选省高水平大学（高职）建设单位，天津大学—密西根大学（南通）联合研究院成立。深化综合医改。推进分级诊疗，组建五大专科联盟、市区紧密型医联体和13个县域医疗集团，推行家庭医生签约服务。实施市属公立医院绩效考核和总会计师制度。与上海交大仁济医院开展合作。提升文体惠民能力。深化公共文化服务体系建设，群众性文化活动和全民阅读扎实开展，话剧《张謇》、越剧《仁医寸心》获好评，10部作品获省“五个一”工程奖。成功举办市第十一届运动会暨第二届全民健身运动会。妇女、儿童、青少年、关心下一代、老龄、残疾人等事业长足发展。完善社会治理体系。落实综治、法治、信访领导责任制，开展社会矛盾和安全隐患大排查大整治，提升立体化、智能化社会治安防控水平，完善城乡社区网格化服务管理体系，市县镇村四级综治中心全覆盖，公众安全感、法治满意度继续位居全省前列。加强安全生产责任落实与隐患治理。推进国家食品安全示范城市创建。实现全国文明城市“四连冠”，如皋成为全省江北首个县级全国文明城市。推动军民融合创新示范区创建，民族、宗教以及国家安全等工作取得新成效。落实扶贫协作和对口支援任务，全域结对帮扶陕西汉中10个县、区。

　　（七）加快转变职能，从严治政更为扎实。把全面从严治党贯穿政府工作全过程，推动“两学一做”学习教育常态化制度化，深入开展富民强企奔小康“走帮服”活动。实施“不见面审批（服务）”改革，优化集中审批、联合审图、代办帮办等机制，建成政务服务“一张网”，服务事项90%可网上办，实现“3550”目标，首轮“双创”环境评价全省第一。启动承担行政职能事业单位改革，推进文化、城管领域综合执法体制改革，全面推广“双随机一公开”。落实法治政府建设实施方案，健全重大行政决策程序规定，开展行政执法三项制度试点。实施城市建筑垃圾管理、水利工程管理条例。认真办好市人大代表议案、市政协建议案，完成市人大代表建议、政协提案办理任务。严格落实党风廉政建设“两个责任”，构建政府投资工程建设领域阳光防范体系，推进公共资源交易市县一体化，行政监察和审计监督全面加强。

　　各位代表，过去一年的工作成绩凝聚着全市人民的辛劳和智慧，得益于方方面面的努力和支持。在这里，我代表市人民政府，向全市人民，向人大代表、政协委员和离退休老同志，向市各民主党派、工商联、无党派人士、各人民团体和各界人士，向驻通人民解放军、武警官兵和公安干警及部省驻通单位，向关心支持南通发展的海内外朋友，致以崇高敬意和衷心感谢！

　　立足新的方位和坐标审视，工作中不少短板和不足时时警醒着我们。一是对照经济发展不平衡不充分的问题，我市在总量、均量、质量上都有待提升，产业结构总体处于中低端，特色产业、龙头企业不多，科技创新能力不强；城市化水平相对偏低，中心城市首位度、人口吸引力有待提高，城乡差距仍较明显。二是对照高水平全面建成小康社会的省标和民标，居民人均可支配收入、全社会劳动生产率仍低于全省平均水平，公共服务有效覆盖面还不够，优质教育、医疗、文化等资源供给不均衡。三是对照生态文明建设的新要求，水、大气、土壤污染防治形势严峻，快速发展中积累的生态环境问题成为明显短板，群众反映比较强烈，生态产品供给与需求矛盾加剧。四是对照防范重大风险面临的挑战，硬化财政预算约束的意识还要增强，管理规范、风险可控的政府举债融资机制还不完善；公共安全基础还不牢固，安全生产责任体系还不严密。五是对照形式主义、官僚主义的种种表现，一些公职人员法治意识、责任意识、服务意识还不强，少数干部懒政怠政、推诿扯皮甚至贪污腐败。我们必须直面问题，采取切实有效措施，认真予以解决。

　　二、准确把握2018年工作的总体要求

　　今年是贯彻党的十九大精神的开局之年，是改革开放40周年、决胜全面建成小康社会的关键一年，也是南通抢抓多重国家和区域战略叠加机遇，加快建设上海“北大门”的重要一年。随着“一带一路”建设、长江经济带发展和省“1+3”功能区战略深入实施，长三角城市群、扬子江城市群规划加快落地，南通站到了新时代发展的“风口”。同时，国际经济和区域竞争格局正加快重构，新旧动能转换乏力，资源环境约束凸显，南通也处在爬坡过坎的关键时期。我们要坚定信心、增创优势，迎难而上、砥砺前行，奋力推动各项工作迈上新台阶。

　　做好今年政府工作，要全面贯彻党的十九大精神，以习近平新时代中国特色社会主义思想为指导，认真落实总书记视察江苏重要讲话精神和中央经济工作会议精神，按照省委、市委全会部署，紧扣社会主要矛盾变化，围绕在高质量发展上走在全省前列的目标，统筹推进“五位一体”总体布局和协调推进“四个全面”战略布局，做好稳增长、促改革、调结构、惠民生、优生态、防风险各项工作，重点推动产业、城市、交通转型升级，打造创新、改革、开放动力引擎，加快民生福祉、生态质量、社会治理改善提升，打好“三大攻坚战”，推进“两聚一高”新实践，加快建设长三角北翼经济中心、全国性综合交通枢纽、具有较强区域影响力的创新之都、宜居宜业富有魅力的花园城市，推动“强富美高”新南通建设取得更大进展。

　　建议今年全市发展的主要预期目标为：地区生产总值增长8%左右，一般公共预算收入超过600亿元，全社会研发投入占地区生产总值比重2.74%左右；固定资产投资增长8%以上，高新技术产业投资增长10%左右，工业投资、技改投资分别增长8%、10%左右，社会消费品零售总额增长10%以上，进出口总额增长6%以上；城乡居民收入增长与经济增长同步，居民消费价格涨幅3%左右；城镇登记失业率控制在3%以内、新增就业8万人以上；节能减排完成省下达目标任务，大气、水环境质量持续改善。

　　工作中，要紧扣新时代要求，更加注重做到：

　　（一）始终把学习贯彻党的十九大精神作为首要政治任务。自觉用习近平新时代中国特色社会主义思想武装头脑、统一思想、指导实践，全面贯彻基本理论、基本路线、基本方略，提高系统化思维、项目化实施、精准化落实的能力，一步一个脚印，踏踏实实干好工作，以接续奋斗的定力、敢于攻坚的担当、务实扎实的行动，把党的十九大描绘的宏伟蓝图转化为生动现实，在谱写中国梦南通篇章中展现新气象新作为。

　　（二）始终把握稳中求进工作总基调。科学把握工作的策略和方法、节奏和力度，正确处理“有守”与“有为”的关系，聚力打好防范化解重大风险、精准脱贫、污染防治攻坚战，全力守住经济持续健康发展的底线、社会大局安定和谐的底线、人民生活和生态环境质量持续改善的底线，着力在转换增长动力、推动改革突破、拓展发展空间等方面抢占先机，着眼长远、立足当前、稳扎稳打，全力保持经济社会发展良好势头。

　　（三）始终坚持高质量发展的根本要求。坚定不移落实新发展理念，坚持质量第一、效益优先，以供给侧结构性改革为主线，推动质量变革、效率变革、动力变革，抓牢项目建设“牛鼻子”，打造实体经济、科技创新、现代金融、人力资源协同发展的现代产业高地，让创新成为第一动力、协调成为内生特点、绿色成为普遍形态、开放成为必由之路、共享成为根本目的，努力实现更高质量、更有效率、更加公平、更可持续的发展。

　　（四）始终践行以人民为中心的发展思想。把满足人民日益增长的美好生活需要作为奋斗目标，抓住老百姓最急最忧最怨的事情，尽力而为、量力而行，积极解决群众最关心最直接最现实的利益问题，富“口袋”与富“脑袋”结合，优化公共服务与丰富精神生活并举，关注弱势群体与促进公平正义同步，持续增进民生福祉，不断增强人民获得感、幸福感、安全感，不让一个人在全面建成小康社会、推动共同富裕的道路上掉队。

　　各位代表，为者常成，行者常至。我们将不忘初心，牢记使命，以决胜全面建成小康社会的实际行动，推动习近平新时代中国特色社会主义思想在南通落地生根！

　　三、落实高质量发展要求，抓好2018年重点工作

　　按照中央、省委省政府和市委部署要求，围绕高质量发展，在抓重点、补短板、强弱项、增优势上下功夫，做好八方面工作：

　　（一）强攻产业转型升级，促进实体经济提质增效。以建设现代化经济体系为目标，加大转方式调结构力度。

　　做强先进制造业。积极创建中国制造2025国家级示范区，加快发展“3+3+N”重点产业。推动船舶海工向特种船舶、智能船舶和豪华邮轮转型，提升现代家纺品质，壮大光通信、集成电路、新材料、新能源汽车等产业集群，加快发展装配式建筑、绿色建筑和现代电力装备等特色产业，培育生物医药、节能环保等战略性新兴产业。深化“互联网+先进制造业”融合创新，新增市级以上智能车间（工厂）20个。推进军民融合创新示范区创建，打造军民融合产业集群。着力破除无效供给，加大落后产能淘汰力度，依法依规推进化工、印染、钢丝绳等落后低效产能退出，加快城镇人口密集区危化品生产企业搬迁。

　　培育新经济新动能。推动互联网、大数据、人工智能和实体经济深度融合，建设新一代信息技术产业“一核两区六基地”。聚焦数字经济、共享经济、生物经济和现代供应链等领域，壮大新产品新业态新模式。加快发展现代物流、金融、服务外包等现代服务业，培育电子商务、研发设计、数据服务等服务业创新平台。拓展全域旅游、医养结合、教育培训等中高端消费，增加品质、服务、绿色消费供给，培育省级品牌消费集聚区5个。

　　加大有效投入力度。突出抓好省级重大项目和市级“双百工程”项目，完成投入超1000亿元。实施重大产业项目突破工程，整合设立重大产业项目发展资金，健全10亿元以上重特大项目市县联动推进机制，引进50亿元以上项目不少于10个。更加注重抓好项目竣工与转化，新增规模以上工业企业300家以上。落实进一步激发社会领域投资活力“37条”，民间投资增长10%以上。

　　振兴实体经济企业。实施“5215”工业大企业培育计划和企业上市三年行动计划，发挥陆海统筹发展基金作用，支持产业链整合和企业并购，50亿元以上销售工业企业达22家。提高金融服务能力，丰富现代金融产品，用好转贷基金，优化中小企业金融服务平台。落实降成本措施，重点降低企业制度性交易、税费负担、生产要素等成本。贯彻《中小企业促进法》，完善“张謇杯”杰出企业家和“三名”人物激励机制，创新“南通企业家日”活动内容和方式，营造企业家健康成长环境，传承优秀企业家精神，调动企业家积极性、主动性、创造性。弘扬劳模精神和工匠精神。推进质量提升和“标准化+”，制订、修订国家和行业标准50项。

　　（二）加快创新之都建设，持续提升科技创新竞争力。优化创新生态，提高创新浓度，科技进步贡献率达63%。

　　强化企业创新主体作用。实施创新型企业培强扶优行动，培育科技“小巨人”和“独角兽”企业，高新技术产业产值占规模以上工业比重达49.5%。支持规模以上工业企业增加研发投入，推动普遍建立研发准备金制度，新增国家级企业技术中心1家、省级以上研发机构20家。优化市级科技发展资金投向，引导企业加强应用研究、集成创新，突破收益分配和股权激励、技术转移开发等障碍，实施20个市级以上重大科技成果转化项目。

　　提升创新载体和平台功能。加快中央创新区科创中心一期和大学科创园建设，实施“5+5”重大科技创新平台建设工程。推动高新区在全省综合评价中争先进位，支持有条件的地区创建省级高新区。推进科技平台“十百千”工程，建设光电工程、先进通信、新材料等重大创新平台，新建和提升研发设计、先进封测、试验认证等公共服务平台。做强1-2个细分行业的产业创新中心，支持共建产业技术创新联盟，推动“两院”院士在企业、高校交叉建立工作站。推进规上高企产学研全覆盖，实施合作项目500个。发挥科技金融撬动创新的作用，探索投贷联动、投保联动、投债联动，扩大“苏科贷”“通科贷”规模。创建国家知识产权强市，每万人发明专利拥有量达25.5件。

　　集聚创新创业人才。落实人才发展促进条例，实施高端人才引领、青年人才倍增和通籍英才归雁计划，办好江海英才创业周暨青年人才发展大会，举全市之力引进1-2个具有重大原创技术的创新创业团队，新增省“双创”人才40名。优化人力资源配置，壮大知识型、技能型、创新型劳动者队伍。以国家级“双创”示范基地为目标，规划建设10个左右的众创社区，打造创新创业全过程服务链，提升“创新南通”平台功能。

　　（三）抢抓重大战略机遇，构建区域协调发展新格局。策应国家、省新一轮开放大布局，以落实扬子江城市群规划为契机，推进跨江协同、江海联动，加快对接上海、走向世界。

　　推动上海“北大门”建设。编制《南通·上海大都市北翼门户城市战略规划》，落实各项工作推进方案，举办对接服务上海大会。打造“南通成本、上海优势”品牌，构建“一核十区多园”的先进制造业协同发展布局。建立“1+3”离岸创新和技术集聚转移平台，争当上海新一轮科技资源转移承接地。提升濠河、狼山、圆陀角等龙头景区功能，精心建设旅游度假区、郊野公园、旅游风情小镇。建设10个优质农副产品保障示范基地，农产品在沪销售额超过150亿元。加强与上海共建崇明世界级生态岛和长江口生态保护战略协同区。深化公共服务同城化，丰富教育、医疗和文化等领域合作。推动通州湾临港产业城打造“一带一路”合作开发园区，支持启东生命健康科技城加快规划建设。

　　打造综合交通枢纽。以交通引领沪通同城化，加快沪通铁路（沪通长江大桥）、盐通铁路、宁启铁路二期建设，深化北沿江高铁、通苏嘉城际前期工作。推进海启高速、锡通高速北接线工程，力争绕城高速、宁通高速扩容开工。开辟国际国内客货运航线。开展加密过江通道、市域铁路、机场客运枢纽规划研究。加快通海港区集装箱码头、横港沙现代化散货码头和沿海港区航道建设。扩容沪通干线传输网络，IP城域网出口带宽达8000G。

　　勇当沿海经济带新增长极。制定发展现代海洋经济工作方案，推进国家海洋经济创新发展示范项目，海洋生产总值增长9%左右。加快沿海重特大项目建设，打造海洋生物医药与南极磷虾、海洋高端装备和能源装备三大产业基地，支持洋口港建设能源交割中心，发展远洋捕捞、海产品深加工和滨海旅游业。推进通州湾江海联动示范区高起点开发、高质量发展。着力培育省级以上船舶海工创新中心、海洋经济发展研究院等协同平台。

　　（四）着眼人的城镇化，提升城乡建设治理水平。切实提高城镇人口素质和居民生活质量，提升中心城市首位度，促进“四化”同步发展。

　　加快城市提质转型。着力做大做强做美做优城市，营造精致宜居的品质环境。提高规划科学化水平。推进2035城市总体规划编制改革，以主体功能区规划为基础统筹各类空间规划，建设“多规合一”信息平台。编制形成“城市双修”专项规划、城市设计总体规划，坚持“留白增绿”，推进生态修复、城市修补和海绵城市建设，加强天际线和色彩管控，提升建筑品质，彰显江风海韵的城市特色。加快“一主五片两重点”建设。推动主城区有机更新，加快棚户区改造、老小区整治、危房解危。提升中央创新区专项规划与建设水平，大力实施基础设施和功能性项目。加快五山国家森林公园建设和5A级景区创建，推进沿江片区生态修复。推动平潮高铁西站片区综合开发建设。支持通州区与主城区基本公共服务一体化。推进257项城建工程，完成“三城同创”三年行动计划。推动轨道交通1号线一期工程建设，力争2号线一期工程开工。增强治理和服务能力。开展城市环境“八整治八提升”行动，着力化解市民反映强烈的违章搭建、餐饮油烟污染、物业管理等突出问题，深化垃圾分类和治理。推进城市“微更新”，畅通“微循环”，优化“微服务”。保障城市公共安全。落实公交优先行动计划。制定和实施“厕所革命”工作方案。

　　实施乡村振兴战略。按照产业兴旺、生态宜居、乡风文明、治理有效、生活富裕的要求，推进农业农村现代化。坚持质量兴农、绿色兴农，调优种植业结构，新增高效设施农（渔）业7.2万亩。提升粮食安全保障水平，加强“菜篮子”工程建设。实施科技支农强农富农工程，推进粮食生产全程机械化、农业科技和信息进村入户。推动高标准农田、适度规模经营、社会化服务联盟“三个全覆盖”试点镇建设，新建高标准农田45万亩，新增市级以上示范家庭农（渔）场34家，建成农民合作社综合社50个。促进农村一二三产融合发展，推广“一村一品一店”，培育100个休闲农业精品景点。推进特色小镇健康发展，抓好特色田园乡村省级试点，培育10个市级示范村庄。振兴乡村文化，推动“乡村记忆影像志”工程成为留住乡愁、传播文化的载体。

　　夯实城乡协调基础。深化新型城镇化试点和经济发达镇改革试点，优化城镇空间布局，进一步放宽重点群体落户条件，常住人口城镇化率达66.6%以上。完善承包地“三权”分置制度，加强农村产权流转交易市场规范化建设。全面启动农村集体产权制度改革，有序推进经营性资产股份合作制改革。推进“四好农村路”、农村重点水利工程和农桥改造项目建设。开展农村人居环境整治三年行动，完善长效管理机制。健全乡村治理体系，培养懂农业、爱农村、爱农民的“三农”工作队伍。

　　（五）激发内生动力，推动改革开放重点任务落地。以深化改革、扩大开放、创新机制为重点，释放和集聚新动能。

　　深化陆海统筹发展综合改革。以第三次全国土地调查为契机，全面落实耕地保护责任，鼓励低效用地再开发、工业用地先租后让，建立海域使用权不动产统一登记制度。落实新一轮财政管理体制，抓好环保税开征和税收征管现代化。积极实施金融改革创新，壮大地方法人金融机构，推进专业金融集聚区建设。完善政府投融资决策机制，严格“借、用、还”闭环管理，依法开展存量债务置换，严格管控新增债务，杜绝违法违规融资行为，切实降低地方债务水平，有效防范化解政府债务风险。深化国资国企改革，加快国有融资平台公司市场化转型，完善国资监管体系，促进国有企业做强做优做大。加强价格机制改革。

　　大力促进双向开放。推进外贸强市建设，放大市场采购贸易方式试点效应，争创跨境电子商务综合试验区，建设外贸转型示范区、进出口基地和外贸集聚区，大力发展服务贸易。加快口岸功能建设，推进综保区与通海港区区港联动。实施外资“植根南通”行动，坚持引资和引技引智并举，加大产业链招商力度，引导现有外资企业增资扩股，力争注册外资实际到账25亿美元。加强与“一带一路”沿线国家和地区经贸合作，支持有条件的企业布局全球创新网络，建设境外产业集聚区，发展国际工程总承包，提升国际货运班列水平。深度融入长江经济带建设，推进以港口为重点的多式联运，深化与长江中上游地区合作。

　　推进开发园区创新转型。全面落实“六大工程”和政策意见，以“一县一园区一特色”为导向，有序推进园区整合优化，提升投资强度和产出效益。省级以上开发园区按照“产业集聚、企业集中、功能集成”的要求，提高核心区规划建设水平，重点打造1个主导产业、扶持壮大1个战略性新兴产业，培育一批500亿级以上特色产业集群。创新开发园区体制机制，扩大区域能评环评改革试点，深入实施全链条审批赋权清单管理。

　　（六）打好污染防治攻坚战，加快建设美丽南通。牢记只有恢复绿水青山才有金山银山，坚决不要“沾着灰”“带着毒”的GDP，下更大的力气补齐生态环境突出短板。

　　攻坚污染大整治。持续抓好中央环保督察整改“回头看”，以最坚决的态度、最有力的措施整改国家海洋督察和省环保督察反馈问题，以接受长江经济带生态环境保护审计为契机，挂牌督办区域性、流域性、行业性问题，坚决不容得过且过、虚假整改，全力攻坚污染防治十大专项治理。治理大气污染。“控煤、降尘、治企、管车、禁烧、联防”并举，推进重点行业挥发性有机物治理，加强工地扬尘、船舶污染等综合整治，PM2.5平均浓度不高于40微克/立方米，空气质量优良天数比例不低于72.5%。治理区域水环境。完善“河长制”和“断面长制”，落实生态河湖行动计划，深入推进河岸共治和“三河三行业”整治，强化16条骨干河道和城乡黑臭水体治理，非禁养区规模化畜禽养殖场治理率达75%，严控水产养殖污染，地表水省控断面优Ⅲ比例达67.7%。按照区域治污的理念，统筹环境基础设施规划建设，重点推进乡镇污水收集管网建设。治理土壤和固体废弃物污染。推进土壤污染详查和修复，建立污染地块名录和开发利用负面清单。加快工业产废处废规范化建设，危废年处置能力达32万吨以上。

　　创新治理大体制。坚持政府为主导、企业为主体、社会组织和公众共同参与，完善全面、全域、全民治理环境的体制。成立污染防治攻坚指挥部，整合市、县、镇、村四级力量，形成齐抓共管、强力推进格局。严格执行生态环境保护工作责任规定，建立督查考评机制，层层压实压紧污染防治责任。全面落实生态环境损害赔偿制度，实施工业企业资源利用绩效综合评价，落实差别化环境政策，发展绿色金融，完善环保信用制度，推动重点排污单位设立环保总监，督促企业把环保责任落到实处。健全行政督察、民主督察、舆论督察等相结合的综合督察体系，支持社会组织和公众当好环保“啄木鸟”，让污染无处藏身。

　　构建执法大机制。持续开展环境执法“亮剑行动”，保持“铁腕治污”高压态势。强化刑事司法。充分发挥司法机关职能，公安机关及时立案侦查涉嫌环境污染犯罪案件，提前介入、直接查处重点案件；加强行政执法与刑事司法衔接，推进环境公益诉讼，形成严惩环境污染犯罪的强大震慑。严格行政执法。深入推进环境监察监测执法垂直管理体制改革，推行突击执法、点穴执法、交叉执法。加大“三行业”企业专项执法力度，关停“散乱污”企业250家。深化综合执法。完善“大环保”综合执法机制，整合各方执法力量，加大联合执法力度，增强打击合力。

　　推动生态大创建。按照国家级生态文明建设示范市标准，推进创建工作。加快海洋生态文明示范区建设，加强入海河流和近岸海域污染防治。建设资源循环利用基地，提升园区循环化改造水平。实施长江经济带生态环境保护规划，强化饮用水源地保护和排污口管理，推进沿江重点规划区域化工企业关停并转迁，完成国家级水生态文明城市建设试点任务。完善生态红线区域保护规划，细化落实主体功能区战略和制度，统筹山水林田湖系统治理。完成国家森林城市创建任务，新增造林13.8万亩。

　　（七）大力繁荣江海文化，守护共同的精神家园。坚定文化自信，建设江海特色文化强市，让城市有根有魂、形神兼备。

　　强化核心价值引领。坚持用中国特色社会主义和中国梦凝聚共识，弘扬“包容会通、敢为人先”的新时期南通精神。实施公民道德建设工程，推动县级全国文明城市创建，光大精神文明“南通现象”。推进诚信建设制度化，开展“志愿江海20周年”系列活动。提升公民科学素质，繁荣哲学社会科学。落实意识形态工作责任制，推动全民阅读，营造清朗网络空间。健全社会心理服务体系，培育自尊自信、理性平和、积极向上的社会心态。

　　传承城市历史文脉。深化江海文化研究，充分挖掘利用地方文化资源。提升南通博物苑及环濠河博物馆群文化吸引力，稳妥实施寺街西南营等历史文化街区保护修缮。推进唐闸历史工业城镇申遗工作，扩大南通非物质文化遗产影响力。加强历史地段、文物古迹和古镇古村保护。

　　发展文化事业产业。完善公共文化服务体系，加快南通大剧院、美术馆等重大文化设施建设，推动基层综合性文化服务中心全面达标。针对不同群体需求，丰富群众性文化活动。深化文化事业单位改革，激发文艺创作活力，推出地域题材、百姓视角的精品力作，讲好“南通故事”。实施文化产业重点项目50个以上，推进重点文化产业示范基地建设，文化产业增加值占地区生产总值比重达5.2%。推动文化与体育、科技、金融等深度融合，培育创意设计、信息服务、休闲旅游等新业态。广泛开展全民健身活动，备战第十九届省运会，实施新一轮足球改革发展三年行动，创建全国青少年校园足球改革试验区。

　　（八）始终突出民生优先，更加注重共建共享。落实“七个有”要求，加大富民惠民安民力度，加快形成人人参与、人人尽力、人人享有的生动局面。

　　推动就业增收、脱贫攻坚。深入落实富民增收“五大机制”“五大行动”和50条政策措施。实施更加积极的就业政策，扩大重点群体就业空间，强化职业技能培训，加强对灵活就业、新就业形态的扶持。实施留学回国人员创业、大学生创业引领、农村创业富民、城镇失业人员创业四项计划，提升创业服务基地功能，支持1.2万名以上群众创业。构建和谐劳动关系，推进工资集体协商。针对不同致贫原因精准扶贫，抓好92个经济薄弱村扶贫项目建设，年收入7000元以下建档立卡低收入人口脱贫率达80%以上。加大健康扶贫力度，动态解决因病因残致贫。做好与陕西汉中的扶贫协作工作，拓展产业、劳务和人才合作。扎实推进与新疆伊宁、青海贵德的对口援建项目，加强与辽宁辽阳的对口合作。

　　推进公共服务标准化均等化。对照“十三五”时期基层基本公共服务功能配置清单，补好短板，优化供给。促进教育优质均衡多样发展。重视婴幼儿照护和儿童早期教育服务，推进义务教育改薄工程和标准化建设，促进普通高中分类发展，深化产教融合，支持在通高校建设省高水平院校。坚持立德树人，培养高素质教师队伍。普及残疾学生15年免费教育，发展继续教育，支持和规范社会力量兴办教育。推动健康南通建设。深化综合医改，加强紧密型医疗联合体建设，健全现代医院管理、分级诊疗、药品供应保障等制度。创新全科医生培养与使用激励机制，做实家庭医生签约服务。加快基层医疗卫生机构建设，完善急救体系，推动健康大数据平台开放应用。扎实开展计划生育暖心服务。深入实施居家和社区养老服务改革、医养结合两项国家试点。提升社会保障水平。持续推进社保扩面征缴，完善统一的城乡居民基本医疗保险制度和大病保险制度，农村最低生活保障标准提高到人均不低于600元/月。完善照护保险制度，扩大县级试点范围。健全房地产市场平稳健康发展长效机制，加强多层次住房供给和保障。时刻把群众冷暖放在心上，多搞雪中送炭，完善社会救助、福利、慈善和优抚安置等制度，实施残疾人服务关爱工程。积极发展妇女、儿童、青少年、关心下一代、老龄、红十字等事业。

　　营造安居乐业环境。完善党委领导、政府负责、社会协同、公众参与、法治保障的社会治理体制，健全矛盾纠纷多元化解机制，开展“人民满意信访建设年”活动。深化平安法治创建，优化社会治安防控体系，加强公共安全视频监控联网应用，积极防范、依法打击各类违法犯罪。深入推动城乡社区网格化服务管理，加强社会组织建设，融合推进“政社互动”“三社联动”，96.5%的城市社区、91.5%的农村社区达省级和谐社区标准。以安全生产风险管控为核心，以“一图、两单、三卡、八必须”和信息化管控为主线，坚决遏制重特大安全事故。深化国家食品安全示范城市创建，保障群众饮食用药安全。推进“七五”普法规划实施。强化国家安全和双拥、民族、宗教、档案、保密等工作。

　　各位代表，奋斗才能收获幸福，奋斗才能造福人民。我们将一件事情接着一件事情办，一年接着一年干。今年继续坚持为民发展的导向，坚守节用裕民的正道，办好10件50项实事，让全市人民生活更加幸福美满！

　　四、致力建设人民满意的服务型政府

　　全面学习贯彻党的十九大精神，牢固树立“四个意识”，勤勉务实，艰苦奋斗，不断增强政府执行力和公信力，把南通建设成为行政效能最高、营商环境最好的地区之一。

　　（一）“严”字当头强作风。进一步严明责任、严肃纪律、严格管理，让求真务实、追求卓越、清正有为蔚然成风。严在标准质量。强化政府工作目标管理，提升推进、协调、督查实效，凡事力求做到细致、精致、极致，坚决克服低标准、过得去、图应付。严在作风硬朗。坚持敢抓敢管、一抓到底，层层落实责任、传导压力，坚决制止不作为、慢作为、乱作为。持续开展“走帮服”，哪里有困难、有矛盾，就到哪里去解决。严在自身要求。深入贯彻中央八项规定《实施细则》精神、省委《具体办法》和市委要求，力戒形式主义、官僚主义，驰而不息纠正“四风”。扎实开展“不忘初心、牢记使命”主题教育，塑造忠诚干净担当的政府形象。从严落实党风廉政建设责任制，支持行政监察向国家监察转变，发挥政府投资工程建设领域阳光防范体系、“阳光扶贫”监管系统等作用，坚决以零容忍的态度惩治腐败。着力建设高素质专业化干部队伍，落实鼓励激励、容错纠错等机制，倡导全体公职人员夙兴夜寐、激情工作、蓬勃向上。

　　（二）“实”字打底见成效。坚持目标导向、问题导向、结果导向，找准工作落点和抓手，说到做到，做就做好。务项目之实抓发展。坚持不懈怠、不急躁、不掺水的要求，更加注重抓好对转型升级有带动力的大项目好项目，严守环保、产出和技术标准等底线，加快催生新的经济增长点。务突破之实抓改革。健全重点改革任务动态跟踪机制，奔着问题去，盯着问题改，着力突破改革协同、要素配置、运作机制等方面的难点和痛点。务感受之实抓民生。坚持问政于民、问需于民、问计于民，让惠民政策和举措更加贴近群众需求。更多运用市场化、社会化、信息化手段提高服务能力，积极解决群众的操心事、烦心事。

　　（三）“改”字发力优环境。刀刃向内深化“放管服”改革，打造“不见面审批、零缺陷服务、精准化监管”品牌，建设一流营商环境。降低就业创业门槛。加大减权放权力度，推动“市县扁平管理、一层全链审批”，落实市场准入负面清单。巩固“3550”改革成果，推广投资项目信用承诺制、注册登记全程电子化等举措。推行高效便捷的智能化政务服务。建立统一的大数据管理体系和共享交换平台，提升“市民通（我的南通APP）”、拓展“政务通”、探索建设“全视通”，让数据多跑路、群众少跑腿。更好地联系群众、服务企业，推动政务服务“一张网”向基层延伸，完成市县“12345”平台一体化整合升级，进一步解决“办事难”“推绕拖”等问题。维护公平竞争市场秩序。深化综合行政执法体制改革，完善“双随机一公开”机制，推进包容审慎监管。

　　（四）“法”字在心重善治。牢固树立宪法法律至上的理念，落实法治政府建设责任。加强生态环保、城市管理等领域政府立法，健全依法决策机制，推行重大决策目录清单管理，严格政府合同审查和履约管理。全面实施行政执法三项制度，建立行政诉讼法律援助制度，支持行政诉讼集中管辖。依法接受党内监督、人大法律和工作监督、政协民主监督以及国家监察、司法监督，认真落实重大决策出台前报告和通报制度，切实办好市人大代表议案、建议和政协建议案、提案。完善层级监督机制，加大财政、经济责任、绩效等审计监督力度。强化社会监督、舆论监督，扩大重大建设项目、食品药品安全等领域政府信息公开。

　　各位代表，拥抱新时代，唯有强毅力行；肩负新使命，务必坚志赴事。让我们更加紧密地团结在以习近平同志为核心的党中央周围，在省委省政府和市委的坚强领导下，同心同德，苦干实干，为建设“强富美高”新南通、决胜全面建成小康社会、创造属于全市人民的幸福生活和美好明天努力奋斗！
